# Indoor bacterial, fungal and viral species and functional genes in urban and rural schools in Shanxi Province, China–association with asthma, rhinitis and rhinoconjunctivitis in high school students

**DOI:** 10.1186/s40168-021-01091-0

**Published:** 2021-06-12

**Authors:** Xi Fu, Zheyuan Ou, Mei Zhang, Yi Meng, Yanling Li, Jikai Wen, Qiansheng Hu, Xin Zhang, Dan Norbäck, Yiqun Deng, Zhuohui Zhao, Yu Sun

**Affiliations:** 1 Guangdong Provincial Key Laboratory of Protein Function and Regulation in Agricultural Organisms, College of Life Sciences, South China Agricultural University, Guangzhou, Guangdong 510642 People’s Republic of China; 2 School of Public Health, Sun Yat-sen University, Guangzhou, People’s Republic of China; 3 Key Laboratory of Zoonosis of Ministry of Agriculture and Rural Affairs, South China Agricultural University, Guangzhou, Guangdong 510642 People’s Republic of China; 4 Guangdong Laboratory for Lingnan Modern Agriculture, South China Agricultural University, Guangzhou, Guangdong 510642 People’s Republic of China; 5 Institute of Environmental Science, Shanxi University, Taiyuan, People’s Republic of China; 6 Occupational and Environmental Medicine, Department of Medical Science, University Hospital, Uppsala University, 75237 Uppsala, Sweden; 7 Department of Environmental Health, School of Public Health, Fudan University, Key Laboratory of Public Health Safety of the Ministry of Education, NHC Key Laboratory of Health Technology Assessment (Fudan University), Shanghai Typhoon Institute/CMA, Shanghai Key Laboratory of Meteorology and Health, Shanghai, 200030 China

**Keywords:** Asthma, Rhinitis, Shotgun metagenomics, High school students, Urban/rural, China

## Abstract

**Background:**

Studies in developed countries have reported that the prevalence of asthma and rhinitis is higher in urban areas than in rural areas, and this phenomenon is associated with urbanization and changing indoor microbiome exposure. Developing countries such as China have experienced rapid urbanization in past years, but no study has investigated microbiome exposure and urban-rural health effects in these countries.

**Methods:**

Nine high schools from urban and rural areas were randomly selected in Shanxi Province, China, and classroom vacuum dust was collected for shotgun metagenomic sequencing. A self-administered questionnaire was collected from 1332 students for personal information and health data. Three-level logistic regression was performed between microbial richness/abundance/functional pathways and the occurrence of asthma and rhinitis symptoms.

**Results:**

Consistent with developed countries, the prevalence of wheeze and rhinitis was higher in urban areas than in rural areas (p < 0.05). Metagenomic profiling revealed 8302 bacterial, 395 archaeal, 744 fungal, 524 protist and 1103 viral species in classroom dust. Actinobacteria (mean relative abundance 49.7%), Gammaproteobacteria (18.4%) and Alphaproteobacteria (10.0%) were the most abundant bacterial classes. The overall microbiome composition was significantly different between urban and rural schools (p = 0.001, Adonis). Species from Betaproteobactera, Gammaproteobacteria and Bacilli were enriched in urban schools, and species from Actinobacteria and Cyanobacteria were enriched in rural schools. Potential pathogens were present in higher abundance in urban schools than in rural schools (p < 0.05). *Pseudoalteromonas*, *Neospora caninum* and *Microbacterium foliorum* were positively associated with the occurrence of wheeze, rhinitis and rhinoconjunctivitis, and *Brachybacterium* was protectively (negatively) associated with rhinitis (p < 0.01). The abundance of human endocrine and metabolic disease pathways was positively associated with rhinitis (p = 0.008), and butyrate and propionate metabolic genes and pathways were significantly enriched in rural schools (p < 0.005), in line with previous findings that these short-chain fatty acids protect against inflammatory diseases in the human gut.

**Conclusions:**

We conducted the first indoor microbiome survey in urban/rural environments with shotgun metagenomics, and the results revealed high-resolution microbial taxonomic and functional profiling and potential health effects.

**Video abstract**

**Supplementary Information:**

The online version contains supplementary material available at 10.1186/s40168-021-01091-0.

## Introduction

Since World War II, the prevalence of many chronic allergic and inflammatory diseases such as asthma and rhinitis has increased dramatically [1, 2]. The number of asthma and rhinitis patients has been estimated to exceed 350 and 700 million worldwide, and an even higher number of people suffer from various asthmatic symptoms such as shortness of breath or chest tightness [1, 3–5]. The prevalence of asthma and rhinitis may reach a plateau in developed countries in recent years, but a fast increasing trend is still observed in developing countries. For example, the prevalence of doctor-diagnosed asthma increased from 0.91% in 1999 to 6.8% in 2012 for children 1–8 years old in China [6]. The increasing trend of asthma and rhinitis poses a substantial economic and medical burden for individuals and society.

An interesting phenomenon of these chronic respiratory diseases is that the occurrence of the diseases is higher in urban areas than in traditional farm or rural areas [7–11]. For example, doctor-diagnosed asthma and rhinitis for children in the non-farming area was 88% and 172% higher than children in the farming area [11]. Recent progress in culture-independent high-throughput microbiome studies revealed that indoor microbiome exposure was closely related to the development of asthma and rhinitis [7, 12, 13]. Children living on farms are exposed to a broader range of environmental microorganisms than children living in urban areas [7]. Exposure to diverse environmental bacteria, including Actinobacteria, Alphaproteobacteria and Cyanobacteria, may facilitate the maturation of the immune system and reduce allergic and inflammatory diseases such as asthma and rhinitis [13–15]. However, most of the studies were conducted in developed countries such as Germany, Finland and the USA, and no study reported microbiome comparisons between urban and rural areas in developing countries. Many developing countries have experienced a fast urbanization process in the past twenty years, and it is interesting to see the prevalence of asthma and rhinitis and the exposed microbiome in these regions.

Amplicon sequencing of taxonomic marker genes such as the 16S ribosomal RNA gene of bacteria and the internal transcribed spacer (ITS) region of fungi is the standard protocol to characterize the indoor microbiome composition. The second-generation sequencing technique from Illumina is widely used in many indoor microbiome surveys [16–18]. However, due to the short read length limitation, the Illumina sequencing strategy can only produce a partial region of the marker genes, leading to reduced taxonomic resolution [19]. Third-generation sequencing, such as PacBio, can produce a full-length sequence for amplicon genes with a species-level resolution, but functional inference is not reliable [20]. Shotgun metagenomic sequencing is an amplification-free approach to characterize microbial species and a whole catalogue of functional genes. This approach has been widely used in human gut microbiome studies but has been applied in only a few microbiome studies in built environments such as in commercial aircrafts, metros and hospitals [21–24]. However, no health data were collected in these studies, and thus, the health effects of the microbial species, functional genes and metabolic products were unclear.

Shanxi is a province located in the central northern part of China. The capital of Shanxi Province is Taiyuan, an industrial city with approximately four million residents. The prevalence of doctor-diagnosed asthma and rhinitis among pupils was lower in Taiyuan than in megacities of China such as Beijing and Shanghai [25], but the prevalence of asthmatic symptoms such as wheeze and shortness of breath in Taiyuan was comparable with these megacities [6]. The low prevalence of doctor-diagnosed asthma in this region might be due to the unawareness of the disease in society or lack of access to medical services or other protective factors [25, 26]. The prevalence of asthma and rhinitis among adolescents was not reported in Shanxi Province.

In this study, we conducted the first shotgun metagenomic sequencing in urban and rural indoor environments. Nine high schools in Shanxi Province were randomly selected to characterize the bacterial, fungal, archaeal and viral composition. The prevalence of asthma and rhinitis symptoms, including wheeze, shortness of breath and rhinoconjunctivitis, was surveyed in students, and the association between the indoor microbiome and functional genes/pathways and respiratory symptoms was assessed.

## Materials and methods

### Study design and health data collection

In this study, ten high schools, five from urban areas and five from rural areas, were randomly selected in Shanxi Province, China. Urban and rural areas were defined according to the local administrative management system; urban areas have a much higher density of population and traffic than rural areas. Rural schools were located approximately 30–40 km from Taiyuan city, the capital of Shanxi Province, and urban schools were located in Taiyuan city (Figure S1). Rural schools were defined as school numbers 1–5, and urban schools were defined as school numbers 6–10 (Table S1). In each school, 4 or 5 classes were randomly selected to collect vacuum dust for microbiome profiling. Ten samples failed to produce enough high-quality DNA. In total, 33 dust samples from 9 schools (4 rural and 5 urban) were qualified and sequenced by amplification-free shotgun metagenomics. In each class, ~ 40 students were randomly selected to collect the health data, and in total, 1332 students completed the self-administered questionnaire. Vacuum dust and self-administered questionnaires were collected in March 2008. The study was approved by the School Board of Taiyuan city, principals and teachers in each school and Ethics Committee at Fudan University (IRB#08-03-0119), Shanghai, China. All students gave their formal written consent, and the records were kept at Fudan University.

A self-administered questionnaire in Chinese was distributed to all participants to collect personal information, including gender, age, smoking habits, and parental asthma and allergy, as well as health data, including asthma and rhinitis symptoms in the last 12 months. Questions about doctor-diagnosed asthma and asthma symptoms were obtained from the European Community Respiratory Health Survey (ECRHS), and questions about rhinitis and rhinoconjunctivitis were obtained from the International Study of Asthma and Allergies in Childhood (ISAAC) study. The questions were the following:

“Have you had diagnosed asthma by a doctor?”

“Have you had wheezing or whistling in the chest in the last 12 months?”

“Have you had daytime shortness of breath during rest or after exercise in the last 12 months?”

“Have you had a problem with sneezing, or a runny or a blocked nose when you DID NOT have a cold or the flu in the last 12 months?” (rhinitis), and if the answer is yes, “Has this nose problem been accompanied by itchy watery eyes?” (rhinoconjunctivitis).

Students answered the questionnaire at home with the help of their parents, and medical staff from our research group went through the questionnaire. The students had no information regarding the sampling and data collected in the classrooms when answering the questionnaire.

### Vacuum dust sampling, DNA extraction and shotgun metagenomics sequencing

Dust in a classroom was collected by a vacuum cleaner (400 W) equipped with a dust sampler and a Millipore filter (ALK Abello, Copenhagen, Denmark). The Millipore filter was made of cellulose acetate with a pore size of 6 μm, retaining 74% of particles in the 0.3–0.5-μm size range, 81% in 0.5–1.0 μm, 95% in 1–10 μm and 100% particles in > 10 μm. Each classroom was vacuumed for 4 min, vacuumed for 2 min on the floor and vacuumed for 2 min on the upper surfaces of desks, chairs, bookshelves, teaching platforms and curtain surfaces. The dust samples were sieved through a 0.3-mm mesh screen to obtain fine dust. The fine dust was stored in a − 80 °C freezer until DNA extraction.

DNA extraction and shotgun metagenomic sequencing were conducted at Personal Biotechnology Co., Ltd. (Shanghai, China). Total microbial genomic DNA in dust was extracted by a DNeasy PowerSoil Kit (QIAGEN, Hilden, Germany), following the manufacturer’s instructions. The quality and quantity of the extracted DNA were assessed by agarose gel electrophoresis and a NanoDrop ND-1000 spectrophotometer (Thermo Fisher Scientific, Waltham, MA, USA). The qualified DNA was processed to construct the shotgun metagenomics sequencing library by a TruSeq DNA Nano High Throughput Library Preparation Kit (Illumina, San Diego, CA, USA). The sequencing strategy was paired-end 150 bp reads with an insert size of 400 bp. A dual indexed barcode structure was applied for multiplexing, and 1% PhiX Control v3 was added into the library for quality monitoring. The prepared libraries were stored at – 20 °C before sequencing. The sequencing platform was Illumina HiSeq X-ten (Illumina, San Diego, CA, USA). The cluster density was in the range of 1255–1412 K clusters/mm^2^, and the error rate was < 0.05% for the sequencing run.

### Metagenomics data assembly and analyses

Raw sequenced reads were first processed to obtain high-quality clean reads. Adapter sequences were removed by Cutadapt (v1.2.1) [27], and raw reads were processed by a 5-bp sliding window to trim low-quality sequences (< Q20, read accuracy < 99%). Trimmed reads with length > 50 bp and no ambiguous bases were kept for further analyses. Human reads were removed by KneadData (v0.9.0) and BMTagger (v3.101). The processed clean reads were deposited in the Genome Sequence Archive (https://bigd.big.ac.cn/gsa) in the National Genomic Data Center, Beijing Institute of Genomics with the accession number CRA003476 [28, 29]. The clean reads were assembled by MEGAHIT (v1.0.5) with a succinct de Bruijn graph approach [30]. The coding sequences (CDS, > 300 bp) were predicted by MetaGeneMark (v3.25) [31]. CDSs were clustered by CD-HIT (v4.8.1) [32] at 90% amino acid sequence identity to obtain a non-redundant gene catalogue. The abundance of genes was calculated as the number of aligned reads by SOAPdenovo2 (v1.0) [33]. The taxonomy was annotated by searching against the NCBI-NT database by BLASTN (e value < 0.001) and annotated by MEGAN with the lowest common ancestor approach [34]. The functional gene was annotated by searching the sequence of the non-redundant genes against the KEGG databases (release 90.0) by DIAMOND protein aligner (v2.0.4) with e value < 0.001 and coverage ratio > 40% [35]. LEfSe (linear discriminant analysis effect size) analyses [36] were analyzed on the Galaxy website (http://huttenhower.sph.harvard.edu/galaxy/, v1.0) for the characteristic microbial taxa and functional genes/pathways in urban and rural schools. Microbial compositional variation (beta diversity) was calculated by Bray-Curtis distance metrics and visualized by non-metric multidimensional scaling (NMDS) hierarchical clustering [37, 38]. Permutation analysis (10,000 permutations) was conducted for microbial taxonomic and functional composition between urban and rural samples by the Adonis function in R (v3.6.1). The growth rate of high abundance bacteria was calculated by GRiD with – c = 0.2 [39]. Twenty-two species were included in the GRiD analysis with the following criteria: species relative abundance > 0.5%, taxa annotation resolved at the species level with a high-quality reference, species coverage > 0.2 and low species heterogeneity < 0.3. Reference genomes were downloaded from the prokaryote database of the NCBI genome browser (https://www.ncbi.nlm.nih.gov/genome/browse#!/prokaryotes/). If multiple reference genomes were available, the genome with the smallest number of scaffolds was chosen. The Quantitative Insights Into Microbial Ecology (QIIME, v1.8.0) pipeline and R (v3.6.1) were used throughout the study for data processing, analysis and visualization [40].

### Association analysis between microbial diversity/abundance and symptoms

Three-level (class and school as second and third level) logistic regression was calculated between microbial richness and asthma and rhinitis symptoms by StataSE 15.0 (StataCorp LLC). Current smoking, gender and parental asthma and allergies were adjusted in the regression model. The microbial richness was represented as the number of observed species in major microbial lineages, including the domains Bacteria, Archaea, Eukaryota and Viruses, the kingdom Fungi and Protista and the major taxonomic classes. The associations between the relative abundance of microbial species and KEGG functional genes/pathways and asthma and rhinitis symptoms were also calculated by three-level logistic regression with the same adjustments. To reduce the number of multiple comparisons, we tested only microbial species and KEGG pathways differentially present in urban and rural schools (LDA score > 2) in the regression analyses. Associations with a p value < 0.01 were considered significant results throughout the study, and the false discovery rate (FDR) was also calculated by the p.adjust function with the Benjamini-Hochberg procedure in R (v3.6.1).

## Results

### Prevalence of asthma and rhinitis symptoms

In this study, self-administered questionnaires were collected from 1332 students in the selected classrooms to assess the prevalence of asthma and rhinitis symptoms. All students were Chinese. The students were aged from 15 to 18 years, with a mean age of 16.1 years. A total of 610 students were female (45.8%), and 722 students were male (54.2%). A total of 924 students were from urban schools (69.4%), and 411 students were from rural schools (30.6%). The prevalence of doctor-diagnosed asthma was low in Shanxi (0.9%), and the prevalence in urban schools was higher than in rural schools, with borderline significance (1.2% vs 0.2%, p = 0.09). The prevalence of wheeze was significantly higher in male students than in female students (7.5% vs 4.6%, p = 0.03), and the prevalence of shortness of breath was significantly higher in female students than in male students (28.0% vs 37.7%, p < 0.001). The prevalence of wheeze (7.1% vs 4.1%) and rhinitis (41.9% vs 32.4%) was significantly higher in urban schools than in rural schools (chi-square test, p < 0.05; Table 1). The prevalence of shortness of breath (33.5% vs 30.4%) and rhinoconjunctivitis (15.9% vs 13.3%) was also higher in urban schools than in rural schools but did not reach statistical significance (p > 0.05).

**Table 1:** Prevalence of wheeze, breathlessness, rhinitis and rhinoconjunctivitis among students (N = 1332) in urban and rural high schools in Shanxi, China. p values were calculated by Chi-square test. Significant p values (p < 0.05) were formatted with bold font

Symptoms	Number	Prevalence (%)	Male (%)	Female (%)	*p* value	Urban (%)	Rural (%)	*p* value
Doctor’s diagnosed asthma	12	0.9	1.1	0.5	0.22	1.2	0.2	0.09
Wheeze	83	6.3	7.5	4.6	**0.03**	7.1	4.1	**0.04**
Shortness of breath	432	32.4	28.0	37.7	**< 0.001**	33.5	30.4	0.26
Rhinitis	518	38.9	39.5	38.2	0.63	41.9	32.4	**0.001**
Rhinoconjunctivitis	199	15.1	15.3	14.8	0.82	15.9	13.3	0.22

### Shotgun metagenomics sequencing statistics

The sequenced samples were referred to as “S1-03” (school number 1–class number 3) throughout the study (Tables S2 and S3). In total, 837.6 Gb raw sequence data were produced with 5.6 billion reads. All samples had > 90% nucleotides with base accuracy > 99% (Q20) (Table S2). Human DNA fragments mainly from the skin accounted for a large proportion of our data. A total of 66.2% of reads were removed as having a human annotation, and 261.8 Gb clean microbial data were kept for further analyses (2.2 to 20.8 Gb per sample; Table S2). The maximum scaffold length ranged from 54 kb (S9-04) to 523 kb (S8-04), and the total length of the assembled scaffolds ranged from 2.2 Gb (S10-03) to 3.3 Gb (S10-02; Table S3).

### Microbiome composition in urban and rural schools

**Fig. 1:**
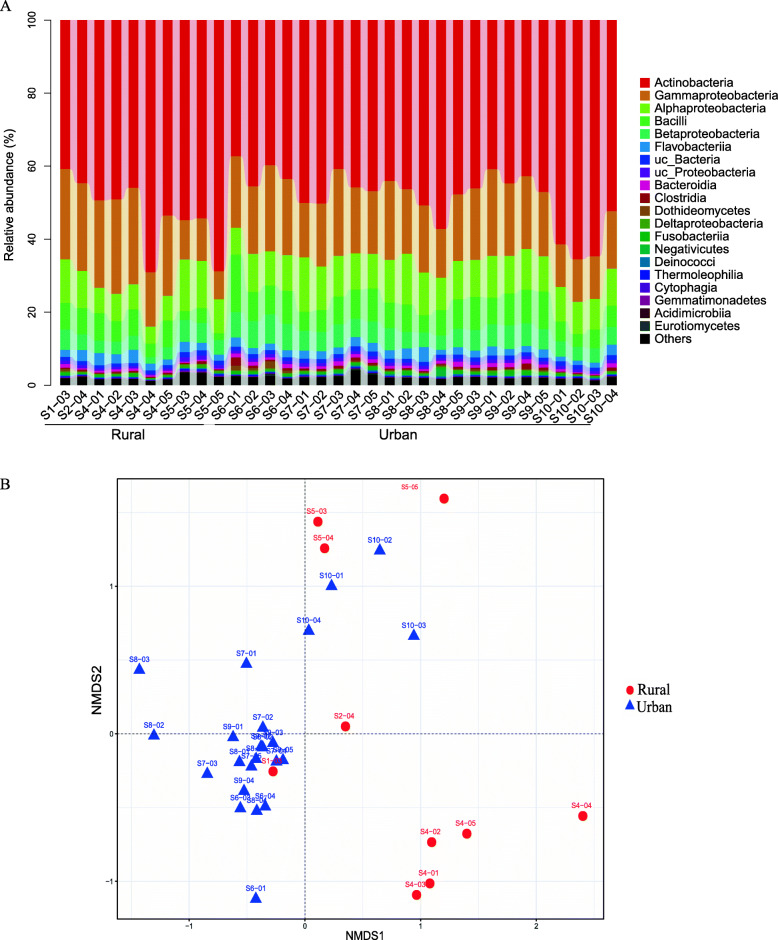
Microbial taxonomic composition and NMDS analysis of taxonomic composition in high schools in Shanxi, China. **A** The taxonomic composition is presented as the relative abundance of major microbial classes. Schools 1–5 are from rural areas, and schools 6–10 are from urban areas. **B** NMDS ordination of the classroom microbiome was calculated based on the Bray-Curtis distance matrix. Ordination plot between axes NMDS1 and NMDS2 is shown

We characterized 11,070 microbial species in this study, including 8302 bacterial, 395 archaeal, 1268 eukaryotic and 1103 viral species. The eukaryotes included 744 fungal and 524 protist species. The plateau curve of the rarefaction analysis indicates that the sequencing depth is deep enough to cover the majority of taxa in the samples (Figure S2). The relative abundances of bacterial, archaeal, eukaryotic and viral taxa were 99.0%, 0.03%, 0.80% and 0.17%, respectively. Actinobacteria (mean relative abundance 49.7%), Gammaproteobacteria (18.4%), Alphaproteobacteria (10.0%), Bacilli (7.0%) and Betaproteobacteria (5.1%) were the most abundant bacterial classes, and Dothideomycetes (0.37%) was the most abundant fungal class (Fig. 1A). The top bacterial, archaeal, fungal, protist and viral taxa are presented in Table 2. The most abundant bacterial species included uc *Actinobacteria* (uc means uncharacterized; 5.1%), uc *Psychrobacter* (3.2%), *Micrococcus luteus* (3.1%), uc *Brachybacterium* (1.8%) and *Janibacter indicus* (1.6%). The highly abundant archaeal species were from the class Nitrososphaeria, including *Candidatus Nitrocosmicus oleophilus* (0.003%) and *Candidatus Nitrosocosmicus exaquare* (0.001%), and the class Halobacteria, including uc *Halobacteria* (0.001%) *and Halorubrum trapanicum* (0.0009%). The most abundant fungal species were common mould species, including *Alternaria alternate* (0.22%), *Alternaria solani* (0.10%), *Aspergillus glaucus* (0.04), *Mucor racemosus* (0.007%) and *Aspergillus aculeatus* (0.005%). The highly abundant protist species were from the class Apicomplexa, including *Neospora caninum* (0.07) and *Babesia bigemina* (0.005%). The highly abundant viruses were mainly bacteriophages, including *Silicibacter phage DSS3phi2* (0.044), *Psychrobacter phage Psymv2* (0.008%) *and Caudovirales phage* (0.004%)*.*

**Table 2:** Relative abundance of the top 20 bacterial, archaeal, eukaryotic and viral species in the classrooms of high schools in Shanxi, China. The species are ordered according to the abundance. “uc” means uncharacterized.

Bacteria	Abundance (%)	Archaea	Abundance (%)	Eukaryota				Virus	Abundance (%)
				Fungi	Abundance (%)	Protista	Abundance (%)		
uc *Actinobacteria*	5.10	*Candidatus Nitrocosmicus oleophilus*	0.0029	*Alternaria alternata*	0.222	*Neospora caninum*	0.065	*Silicibacter phage DSS3phi2*	0.044
uc *Psychrobacter*	3.21	*Candidatus Nitrosocosmicus exaquare*	0.0012	*Alternaria solani*	0.102	*Heteromita globosa*	0.010	uc *Pa6virus*	0.035
*Micrococcus luteus*	3.08	uc *Halobacteria*	0.0011	*Aspergillus glaucus*	0.044	*Babesia bigemina*	0.005	uc *virus*	0.029
uc *Brachybacterium*	1.80	*Halorubrum trapanicum*	0.0009	uc *fungus*	0.013	*Albugo laibachii*	0.005	*Psychrobacter phage Psymv2*	0.008
*Janibacter indicus*	1.59	*Salinigranum rubrum*	0.0008	*Malassezia restricta*	0.009	uc *Pyropia*	0.004	*Caudovirales phage*	0.004
*Kocuria palustris*	1.50	*Haloterrigena turkmenica*	0.0008	*Didymella pinodes*	0.008	*Sterkiella histriomuscorum*	0.003	*Caudovirales phage*	0.004
*Serinicoccus sp.* JLT9	1.32	*Halobacterium hubeiense*	0.0006	*Mucor racemosus*	0.007	*Pyropia haitanensis*	0.003	*Psychrobacter phage pOW20-A*	0.003
*Kocuria turfanensis*	1.27	*Haloferax gibbonsii*	0.0006	*Saccharomyces cerevisiae*	0.006	*Aureococcus anophagefferens*	0.003	*Citrus endogenous pararetrovirus*	0.003
uc *Microbacterium*	1.26	*Salinarchaeum sp.* Harcht-Bsk1	0.0006	*Aspergillus aculeatus*	0.005	*Bodomorpha sp.* HFCC57	0.002	*Alphapapillomavirus 2*	0.002
uc *Xanthomonadaceae*	1.21	*Halorientalis sp.* IM1011	0.0005	*Stemphylium lycopersici*	0.005	*Plasmodium vivax*	0.002	uc *virus*	0.002
uc *Micrococcales*	1.20	uc *Candidatus Nitrosocosmicus*	0.0005	*Parastagonospora nodorum*	0.005	*Salpingoeca rosetta*	0.002	uc *Siphoviridae*	0.002
*Agrococcus carbonis*	1.17	*Halobiforma lacisalsi*	0.0005	*Tremella fuciformis*	0.004	*Emiliania huxleyi*	0.002	uc *virus*	0.001
uc *Paracoccus*	1.13	*Natronococcus occultus*	0.0005	*Pichia kudriavzevii*	0.004	*Plasmodium malariae*	0.002	*Human endogenous retrovirus K*	0.001
uc *Bacteria*	1.12	*Halopiger xanaduensis*	0.0005	*Malassezia globosa*	0.003	*Cavenderia fasciculata*	0.002	uc *virus*	0.001
*Acinetobacter johnsonii*	1.11	*Halovivax ruber*	0.0005	*Ustilago hordei*	0.003	*Colpodella angusta*	0.001	*Sfi21dt1virus*	0.001
uc *Nocardioidaceae*	0.99	*Halopenitus persicus*	0.0005	*Leptosphaeria biglobosa*	0.003	*Plasmodium reichenowi*	0.001	*Cyanobacteria phage AS-1*	0.001
uc *Streptococcus*	0.99	uc *Natrialbaceae*	0.0005	*Aspergillus steynii*	0.003	*Vaucheria litorea*	0.001	*Pa6virus*	0.001
uc *Pseudomonas*	0.95	*Haloarcula taiwanensis*	0.0005	*Diaporthe longicolla*	0.003	*Plasmodium relictum*	0.001	*Alphapapillomavirus 9*	0.001
uc *Nocardioides*	0.85	uc *Archaea*	0.0005	*Alternaria tenuissima*	0.003	*Thecamonas trahens*	0.001	*Mollivirus sibericum*	0.001
uc *Proteobacteria*	0.83	*Halobacterium sp.* DL1	0.0004	*Phoma sp.* 1 OB-2014	0.002	*Plasmodium sp.* DRC-Itaito	0.001	*Pseudomonas virus PB1*	0.001

We further characterized the overall microbiome composition variation by non-metric multidimensional scaling (NMDS) hierarchical clustering. Samples from urban and rural schools were clustered mainly on the left and right sides of NMDS1, respectively (Fig. 1B), indicating that urban and rural classrooms contain different microbial compositions. The variation was also confirmed by the permutation analysis (p = 0.001, Adonis, 10,000 permutations). School 5 showed large compositional variation compared with schools 1–4 along the axes of NMDS2 (Fig. 1B), suggesting that other environmental characteristics were also involved in shaping the variation.

**Fig. 2:**
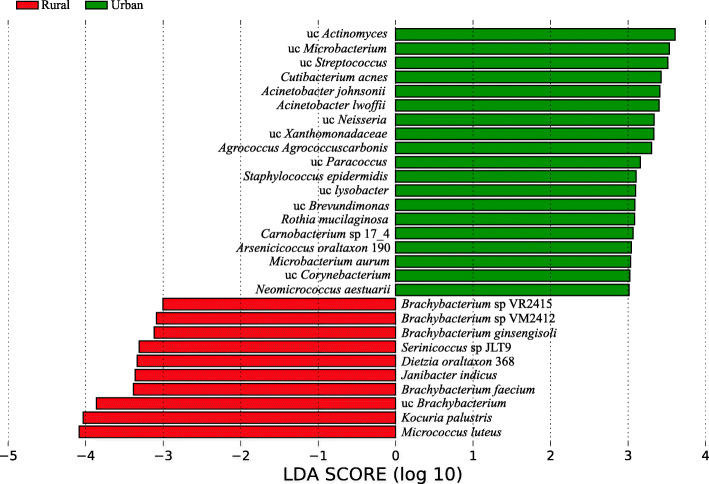
LEfSe analysis for characteristic microbial species in urban and rural schools in Shanxi, China. Only microbial species with LDA score > 3 is shown

To characterize the feature species in urban and rural schools, we conducted LEfSe analysis. Species enriched in urban schools were mainly from classes Betaproteobacteria (uc *Neisseria*), Gammaproteobacteria (*Acinetobacter lwoffi*, uc Xanthomonadaceae, *Acinetobacter johnsonii*, uc *Lysobacter*), Bacilli (uc *Streptococcus*, *Staphylococcus epidermidis*, *Carnobacterium* sp.) and Actinobacteria (uc *Microbacterium*, uc *Actinomyces*, *Cutibacterium acnes*, *Agrococcus carbonis*, *Neomicrococcus aestuarii*; LDA > 3). Species enriched in rural schools were mainly from classes Actinobacteria (*Micrococcus luteus*, *Brachybacterium* sp., *Kocuria palustris*, *Dietzia* sp., *Derinicoccus* sp, *Janibacter indicus*) and Cyanobacteria (Microcoleus sp. *Oscillatoria nigro-viridis*; Fig. 2A).

The potential pathogens defined by NIAID (National Institute of Allergy and Infectious Diseases, USA) were also characterized. Overall, the potential pathogens were present in low abundance (Table S4; total abundance 0.057%). *Clostridium pefringens* (0.021%) and *Listeria monocytogenes* (0.011%) were the top pathogens in the school environment, with other pathogens all < 0.01%. Interestingly, the abundance of pathogens was higher in urban schools than in rural schools (p = 0.046, t test; rural average abundance 0.034%, urban 0.066%). Specifically, *L. monocytogenes*, *Campylobacter jejuni*, *Yersinia pestis* and *Toxoplasma gondii* were present in higher abundance in urban schools than in rural schools (p = 0.001, 0.043, 0.018 and 0.002, t test). *Burkholderia pseudomallei* was the only species present in higher abundance in rural schools than in urban schools (p < 0.001). However, shotgun metagenomics data were assembled in fragmented scaffolds, and thus, the potential health effects of these pathogens should be interpreted with caution.

We also compared the bacterial assemblage of this study to previously reported habitats (Table 3). The bacterial composition in Shanxi high schools was more similar to the air and human skin microbiomes than to the soil, freshwater, human gut and saliva microbiomes (calculated from the Earth Microbiome Project [41]). Additionally, some high-abundance bacterial genera in Shanxi high schools were also present in high abundance in other indoor environments such as the dormitories of Shanxi University, China and junior high school classrooms in Malaysia [12, 42].

**Table 3:** Average relative abundance of the top 15 bacterial genera in high schools in Shanxi and their relative abundance in other habitats. The bacterial abundance in air, non-saline soil and freshwater, and the human gut, saliva and skin environment were calculated from the Earth Microbiome Project [41]. The bacterial abundance in the university dormitory and junior high school was calculated from two recent publications [12, 42]. Taxa with relative abundance > 0.1% were formatted with bold font

Taxa	High school (%)	Air (%)	Soil (%)	Water (%)	Human gut (%)	Human saliva (%)	Human skin (%)	University dormitory, China (%)	Junior high school, Malaysia (%)
*Psychrobacter*	**5.36**	**0.12**	< 0.01	0.01	0	< 0.01	**0.18**	0.04	0
*Kocuria*	**3.82**	< 0.01	< 0.01	< 0.01	< 0.01	0.02	**0.43**	**0.25**	**1.43**
*Brachybacterium*	**3.41**	**0.16**	0.02	< 0.01	0.03	< 0.01	**0.59**	**0.23**	**0.71**
*Micrococcus*	**3.22**	**0.10**	< 0.01	< 0.01	< 0.01	< 0.01	**0.65**	**0.54**	**0.82**
*Acinetobacter*	**2.99**	**2.30**	**0.10**	**1.37**	0.02	**0.21**	**4.93**	**23.2**	**2.21**
*Microbacterium*	**2.93**	0.03	**0.10**	0.06	< 0.01	< 0.01	0.03	**0.75**	0
*Paracoccus*	**2.80**	**0.18**	0.01	**0.13**	< 0.01	< 0.01	**0.32**	**0.13**	**3.13**
*Nocardioides*	**2.55**	0.06	0.03	0.01	0	< 0.01	0.04	0.03	**0.46**
*Streptococcus*	**2.09**	**5.46**	**0.16**	**0.16**	**0.37**	**19.97**	**10.78**	**0.49**	**1.45**
*Pseudomonas*	**2.04**	**1.41**	**1.29**	**2.57**	< 0.01	0.01	**5.44**	**1.88**	**0.80**
*Corynebacterium*	**1.71**	**1.06**	0.04	0.02	**0.89**	**0.43**	**7.66**	**0.95**	**1.77**
*Janibacter*	**1.56**	< 0.01	< 0.01	< 0.01	< 0.01	< 0.01	0.04	0.04	**0.64**
*Planococcus*	**1.50**	< 0.01	0.02	< 0.01	0	0	< 0.01	<0.01	0
*Arthrobacter*	**1.39**	**0.21**	**0.39**	0.04	< 0.01	0.02	0.13	0.09	**0.48**
*Serinicoccus*	**1.32**	0.07	< 0.01	< 0.01	< 0.01	< 0.01	0.04	<0.01	0

### Associations between microbial richness/abundance and asthma and rhinitis symptoms

The associations between microbial richness and asthma and rhinitis symptoms were examined by three-level logistic regression. The prevalence of doctor-diagnosed asthma was very low and was not included in the analysis. The microbial richness in the domains of bacteria, archaea and eukaryotes and the kingdoms of fungi and viruses were not significantly associated with the wheeze, breathlessness, rhinitis and rhinoconjunctivitis (p > 0.05; Tables S5-S8). Similarly, no significant associations were found between the richness of major microbial classes and these symptoms (p > 0.05; Table S5-S8).

The associations between microbial species abundance and wheeze, shortness of breath, rhinitis and rhinoconjunctivitis were examined with the same regression model. To reduce the number of tests, we examined only 117 microbial species differentially present in urban and rural schools (LDA > 2) (Table S9). Five bacteria and one protist were associated with these symptoms. An uncharacterized *Pseudoalteromonas* from the class Gammaproteobacteria was positively associated with wheeze (p = 0.008; Table 4). *Brachybacterium* sp. P6-10-X1 was protectively/negatively associated with rhinitis (p = 0.009), and uncharacterized Betaproteobacteria and *Pseudoalteromonas* were positively associated with rhinitis (p = 0.002 and p < 0.001). Uncharacterized *Pseudoalteromonas* was positively associated with both wheeze and rhinitis. The protist *Neospora caninum* was positively associated with rhinitis (p = 0.002). An uncharacterized Flavobacteriaceae species was negatively associated with rhinoconjunctivitis (p = 0.009), and *Microbacterium foliorum* was positively associated with rhinoconjunctivitis (p = 0.006).

**Table 4:** Association between the abundance of indoor microbial species and wheeze, rhinitis and rhinoconjunctivitis in high schools in Shanxi, China. The β coefficient and 95% confidence interval (CI) were calculated by 3-level logistic regression models adjusted for gender, smoking and parental asthma and allergy. Regression analyses for wheeze and rhinitis were conducted for species differentially present in urban and rural schools (LDA > 2) and mean relative abundance > 0.05%; and thus, 117 species were analyzed. Regression analyses for rhinoconjunctivitis were only conducted for 21 species that were potentially associated with rhinitis (p < 0.05). Only associations with p < 0.01 are presented in this table. The false discovery rate (FDR) was calculated by the Benjamini-Hochberg (BH) procedure. “uc” means uncharacterized

Symptoms	Domain/kingdom	Class	Species	Relative abundance (%)		β (95% CI)	p value	FDR
				Urban	Rural			
Wheeze	Bacteria	Gammaproteobacteria	uc *Pseudoalteromonas*	0.08	0.05	0.11 (0.03, 0.19)	0.008	0.16
Rhinitis	Bacteria	Actinobacteria	*Brachybacterium* sp. P6-10-X1	1.36	2.75	− 0.12 (− 0.21, − 0.03)	0.009	0.17
		Betaproteobacteria	uc Betaproteobacteria	0.10	0.08	0.08 (0.03, 0.13)	0.002	0.08
		Gammaproteobacteria	uc *Pseudoalteromonas*	0.08	0.05	0.06 (0.03, 0.10)	< 0.001	< 0.01
	Protista	Conoidasida	*Neospora caninum*	0.09	0.01	0.03 (0.01, 0.04)	0.002	0.08
Rhinoconjunctivitis	Bacteria	Flavobacteriia	uc Flavobacteriaceae	0.20	0.31	− 0.29 (− 0.51, − 0.07)	0.009	0.09
		Actinobacteria	*Microbacterium foliorum*	0.08	0.06	0.14 (0.04, 0.24)	0.006	0.09

### Abundance of functional genes in urban and rural schools

**Fig. 3:**
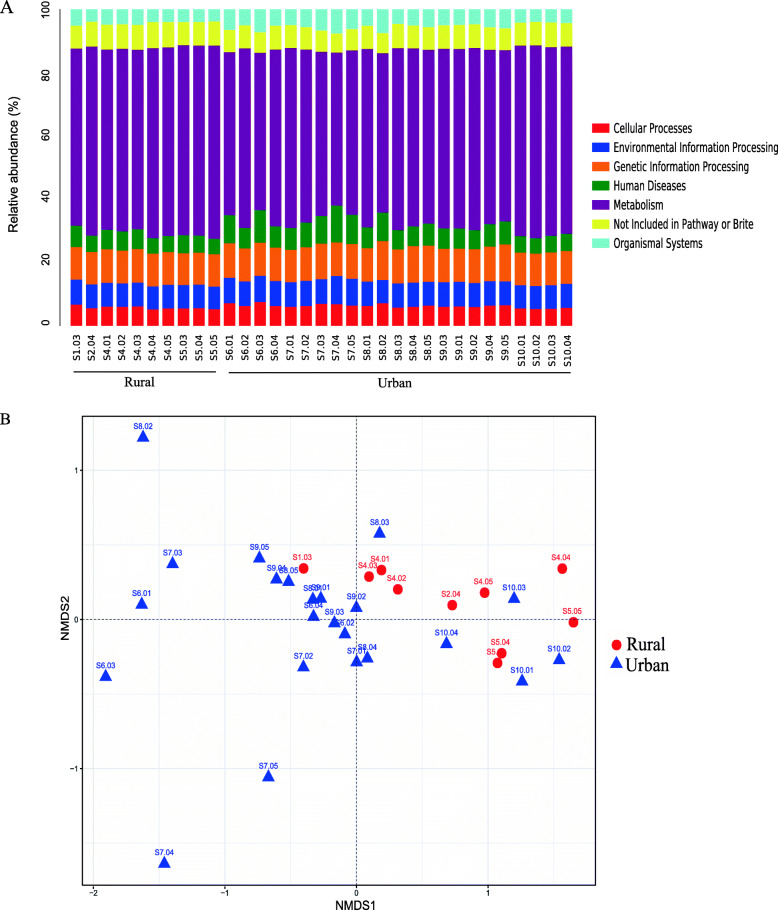
Microbial functional gene composition and NMDS analysis of functional pathways in high schools in Shanxi, China. **A** The relative abundance of first-level KEGG functional pathways. Schools 1–5 are from rural areas, and schools 6–10 are from urban areas. **B** NMDS ordination of classroom functional genes was calculated based on the Bray-Curtis distance matrix. Ordination plot between NMDS1 and NMDS2 is shown

In this study, 15 million non-redundant functional genes were extracted from the shotgun metagenomic assemblies. The functional genes were annotated according to the KEGG Orthology database and classified into different KEGG functional pathways. More than half of the functional genes were annotated as “Metabolism” (56.4%), followed by “Genetic Information Processing” (10.7%), “Environmental Information Processing” (7.7%), “Human Diseases” (6.8%), “Cellular Processes” (6.1%), “Organismal Systems” (5.1%) and “Not included in Pathway” (7.4%; Fig. 3A). Samples from urban and rural schools were also separately clustered in the NMDS analysis (Fig. 3B), and the variation was also supported by the permutation analysis (p = 0.005, Adonis, 10,000 permutations).

**Fig. 4:**
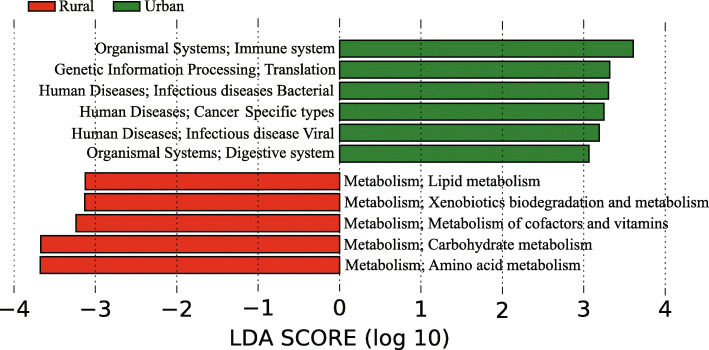
LEfSe analysis for characteristic KEGG functional pathways in urban and rural dust samples in Shanxi, China. The second-level KEGG functional pathways are presented. Only pathways with LDA scores > 3 are presented in the figure

The differential KEGG functional pathways in urban and rural schools were also characterized by LEfSe analysis. Pathways at the second KEGG hierarchical level are shown in Fig. 4. A higher abundance of genes related to human disease and the immune system was detected in urban schools, including “Human disease; Infectious disease Bacterial”, “Human disease; Infectious disease Viral”, “Human disease; Cancers Specific types”, “Organismal Systems: Immune system” and “Organismal Systems: Digestive system” (LDA > 3). A higher abundance of metabolic genes was detected in rural schools, including “Lipid metabolism”, “Amino acid metabolism”, “Carbohydrate metabolism”, “Metabolism of cofactors and vitamins” and “Xenobiotics biodegradation and metabolism” (LDA > 3). A recent study reported that the production of short-chain fatty acids (SCFAs) by gut microbiota, including butyrate and propionate, protected against allergic inflammation in the lungs [43]. Thus, we further conducted LEfSe analysis specific for carbohydrate metabolism at the third KEGG hierarchical level. We found a higher abundance of genes from “Butanoate metabolism” (LDA = 2.7, p = 0.004) and “Propanoate metabolism” (LDA = 2.9, p = 0.002) pathways in rural schools than in urban schools, indicating that more butyrate and propionate may be produced by the indoor microbiome in the rural environment.

The associations between the abundance of KEGG functional genes/pathways and health symptoms were also examined by regression. A higher abundance of the “Human Disease; Endocrine and metabolic diseases” pathway was associated with a higher occurrence of rhinitis (p = 0.008, β = 0.55), but no specific gene was significantly associated with rhinitis (p > 0.01). Genes involved in primary metabolism were protectively associated with rhinitis. Two enzymes involved in butyrate metabolism, including 4-aminobutyrate aminotransferase and diaminobutyrate-2-oxoglutarate transaminase, were protectively associated with rhinitis (p < 0.01), consistent with the LEfSe analysis. Two acyltransferases, including NAD-dependent deacetylase and streptothricin acetyltransferase, were protectively associated with rhinitis (p < 0.01).

### Bacterial growth rate in urban and rural schools

Recent progress in bioinformatics tools enables us to estimate the growth rate of bacterial species by calculating the read coverage in replication origin and terminal regions [39]. Twenty-two species with high read coverage were analyzed. The majority of the species (21 out of 22) had stopped growing or had a very low growth rate (GRiD score < 1.3). *Arthrobacter agilis* was the only species with a GRiD score > 2 (Table S10). Compared with fast-growing species such as *Bdellovibrio* in aquaculture (GRiD score > 5) [39], the indoor species from high schools had a relatively slow growth rate. Additionally, the growth rates of these species were not significantly different in urban and rural schools (p > 0.05, t test). This result indicates that the high microbiome variation between the urban and rural schools is not due to bacterial growth in the indoor environment; rather, it should be due to the variation in the sources of microorganisms, such as outdoor greenness, traffic or other environmental characteristics.

## Discussion

In this study, we conducted the first shotgun metagenomic sequencing in an urban/rural indoor environment and assessed the health effects of microbial exposure. Like Western countries, the prevalence of asthma and rhinitis symptoms was higher in urban areas than in rural areas in China. The overall indoor microbiome taxonomic and functional composition was significantly different between urban and rural schools. Specifically, species enriched in urban schools were mainly from the class Actinobacteria and Cyanobacteria, and species enriched in rural schools were mainly from the class Betaproteobacteria, Gammaproteobacteria, Bacilli and Actinobacteria. Potential NIAID-defined pathogens were present in higher abundance in urban schools. Five bacterial and one protist species were significantly associated with wheeze, rhinitis and rhinoconjunctivitis. Microbial genes in human diseases and immune systems were enriched in urban schools, whereas genes in butanoate and propanoate metabolic pathways were enriched in rural schools. The abundance of genes in the “Human Disease; Endocrine and metabolic diseases” pathway was positively associated with the occurrence of rhinitis.

### Strengths and limitations of the study

This is the first study to reveal urban/rural microbiome exposure and asthma and rhinitis in an Asian developing country, complementing previous observations in Western countries. Another strength is that we applied shotgun metagenomic sequencing to characterize microbiome composition in the school environment. Compared with traditional amplicon sequencing, the technique expands the scope of microbiome detection by characterizing bacteria, archaea, fungi, protists and viruses together with high taxonomic resolution. Additionally, shotgun metagenomics is not subject to PCR amplification biases in 16S rRNA and ITS sequencing, which enables accurate microbial abundance estimation. In addition, the technique enables functional inference, which is not feasible by amplicon sequencing.

A limitation of this study is that the cross-sectional study design restricts causal inference. Additionally, the questionnaire-based study relies on self-assessment of illness or symptoms, but medical staff went through the questionnaire and explained the uncertainty to the participants. The prevalence of doctor-diagnosed asthma was low in Taiyuan (0.9%), and thus, we did not conduct microbial association analysis. The low prevalence is likely due to unawareness of the disease in society and not access to medical services [6, 25, 26]. Unawareness of the disease is probably more common in less developed or rural regions such as Shanxi, than in megacities, such as Shanghai or Beijing. In this study, eleven students in urban schools reported diagnosed asthma, whereas only one student in rural schools reported diagnosed asthma. The numbers were too low to make a solid statistical inference, but it is in line with the idea that unawareness of the disease is more common in rural regions. Unlike diagnosed asthma, the prevalence of wheeze and shortness of breath in Shanxi were comparable or even higher than in developed countries [6, 25]. Thus, it is important to survey asthma symptoms to represent respiratory health in Shanxi. In this study, we conducted a false discovery rate correction (Benjamini-Hochberg procedure) to control type I error and potentially false-positive results in regression [44]. The approach was widely used in genome-wide association studies (GWAS) but was suggested to be overly conservative for microbiome association analysis [45, 46]. A recent algorithm, dsFDR, was proposed to increase the efficiency and power of detection [45]. However, the algorithm does not support multilevel adjustment, which is important in school microbiome studies with several levels of nested data (individual, class, school levels). An alternative solution could relax the standard threshold in the FDR test (< 0.05) to improve the sensitivity of detection. Thus, we reported six potential species associated with asthma and rhinitis symptoms (p < 0.01), and the FDR values were all < 0.2. Finally, metagenomics can provide the functional potential of the microbial community surveyed but does not confirm that the relevant proteins or gene products are expressed and present in the dust. Thus, the functional implications should be treated with caution.

### Microorganisms in urban and rural schools and health implications

We found significantly different microbiome compositions between urban and rural schools in Shanxi. Specifically, urban schools were enriched with species from Betaproteobacteria, Gammaproteobacteria, Bacilli and Actinobacteria, and rural schools were enriched with species from Actinobacteria and Cyanobacteria. The results were consistent with a home study in farm and non-farm areas from Finland and Germany [13]. In that study, the authors reported that Streptococcaceae (a family of Bacilli) was enriched in homes from the non-farm area, and Alphaproteobacteria, Actinobacteria and Cyanobacteria were enriched in homes from the farm area; the latter taxa might provide protective effects against the development of asthma.

In addition to the abundance analysis, a more stringent regression model with multiple adjustments was also applied to examine the potential health-related microorganisms. *Brachybacterium* sp. P6-10-X1 and uncharacterized Flavobacteriaceae were negatively associated with rhinitis and rhinoconjunctivitis. These two taxa are found in a wide variety of outdoor environments, including marine, freshwater, soil, sediment, plants and animals [47, 48], but no study has reported their potential health effects. Three bacteria (uncharacterized *Pseudoalteromonas,* uncharacterized Betaproteobacteria and *Microbacterium foliorum*) and one protist species (*Neospora caninum*) had potential risk effects on wheeze and rhinitis. *Pseudoalteromonas* species are Gram-negative bacteria inhabiting soil, lakes and marine environments [49]. The genus is well-known for its wide application in the pharmaceutical industry. For example, *Pseudoalteromonas phenolica* can produce phenolic substances that suppress the growth of methicillin-resistant Staphylococcus aureus (MRSA) [50]. *Pseudoalteromonas* species can also produce cyclodigiosin hydrochloride, suppressing T-cell proliferation as an immunosuppressant therapeutic agent [51]. Homoeostasis between the type 1 and type 2 immune responses suppresses the development of asthma [14]. Severe immunosuppression in humans over-activates the type 2 response, and prolonged type 2 immunity could increase the risk of atopic sensitization and asthma [52]. Thus, *Pseudoalteromonas* species could provide potential beneficial antibiotic effects and detrimental health effects for asthma. *Microbacterium foliorum* is a Gram-positive bacterium widely used in the production of food ingredients. The species were assessed and found to be non-mutagenic and non-clastogenic in a murine model [53], but no study reported the potential health effects on asthma and rhinitis. *Neospora caninum* can cause abortion and neurologic disease in cattle, and the occurrence of *Neospora* infection is common in dairy herds in the USA [54], but an antibody titer test showed that the species was unlikely to infect humans directly [55]. Additionally, no study has reported the potential health effects of the species on asthma and rhinitis.

In this study, *Brachybacterium* sp. and *Pseudoalteromonas* were associated with rhinitis, and *Microbacterium foliorum* and uncharacterized Flavobacteriaceae were associated with rhinoconjunctivitis. Different species were linked to rhinitis and rhinoconjunctivitis, probably because rhinoconjunctivitis is more specific for allergic rhinitis, while rhinitis also includes non-allergic rhinitis [2, 4, 56].

Another interesting finding of this study is that the potential pathogens were more enriched in urban schools than in rural schools, including *L. monocytogenes*, *Campylobacter jejuni*, *Yersinia pestis* and *Toxoplasma gondii*. This result is consistent with the concept that urbanization may increase infectious diseases [57]. A recent shotgun metagenomics survey also reported that antibiotic-resistant pathogens were commonly detected in the sewage of major cities in China [58], raising awareness of pathogens spreading through the septic system in urban areas.

### Functional genes and health implication

In this study, we found that the genes and pathways related to metabolism were enriched in rural schools and that the pathways related to human diseases and immune systems were enriched in urban schools. The results were supported by a relaxed abundance analysis (LEfSe) and a stringent logistic regression analysis. A study in European countries reported that exposure to rural environments in early life increased the maturation rate of gut microbiota, leading to increased production of short-chain fatty acids (SCFAs), including butyrate and propionate [43]. These SCFAs impair the viability of eosinophils and are particularly important in mediating the protective effect on asthma and inflammatory bowel disease (IBD) [43, 59] [60, 61]. A higher abundance of Bacteroidetes, Alistipes and *Lactobacillus* in the murine gut produced a higher level of butyrate, increasing the expression of MUC2 and intestinal epithelial barrier function and further reducing gastrointestinal inflammation [61, 62]. In this study, we found that SCFAs in the indoor environment may also have anti-inflammatory effects, as in the human gut. The genes and pathways related to butanoate and propanoate production were significantly enriched in rural schools compared with urban schools (p < 0.005). Modern humans spend more than 90% of their time in the indoor environment [63]; numerous microorganisms and their metabolic products in the air can be inhaled into the human body. The elevated production of butyrate and propionate by microorganisms in the indoor environment could provide the same protective effects as the microorganisms inhabiting the human gut. Thus, this finding may provide a new perspective for future prevention and remediation strategies for asthma and rhinitis.

In this study, a higher abundance of genes in the “Human Disease; Endocrine and metabolic diseases” pathway was associated with a higher occurrence of rhinitis. The association between metabolic dysfunction, such as obesity and asthma, is supported by many studies [64, 65]. The prevalence of asthma increases with the body mass index (BMI) of children [66]. Recent progress has also reported that other metabolic dysfunctions also affect the prevalence of asthma. One study surveyed 4000 people in Korea and reported that the number of metabolic dysfunctions, including insulin resistance and systemic inflammation, was positively associated with asthma [67], consistent with our finding. Thus, exposure to microorganisms related to metabolic diseases in the indoor environment may also increase the occurrence of asthma.

## Conclusions

We conducted the first shotgun metagenomics sequencing between urban and rural indoor environments, revealing high-resolution microbial taxonomic and functional profiling and potential health effects. A significantly higher prevalence of asthma and rhinitis symptoms in urban areas compared with rural areas in Shanxi, China. Microbial composition also varied significantly between the two areas, and several potential protective and risk microorganisms were associated with these symptoms. Genes and pathways related to butyrate and propionate metabolism were significantly enriched in rural schools, in line with previous findings that these short-chain fatty acids in the human gut protect against various inflammatory diseases. This study expanded our understanding of the indoor microbiome and respiratory health, providing new insights for indoor microbial exposure from a functional perspective.

## Supplementary Information

**Additional file 1: Figure S1**. Locations of schools sampled in this study.

**Additional file 2: Figure S2**. Rarefaction curve of observed species among all samples.

**Additional file 3: Table S1**. School and sample information in this study. **Table S2**. Sequencing statistics for collected dust samples. **Table S3**. Assembly statistics for collected dust samples. **Table S4**. Relative abundance of NIAID (National Institute of Allergy and Infectious diseases, NIH, US) defined pathogens in urban and rural schools in Shanxi, China. **Table S5**. Associations between microbial α-diversity (observed number of species) and wheeze among students (N=1,332) in high schools, Shanxi, China. **Table S6** Associations between microbial α-diversity (observed number of species) and breathlessness among students (N=1,332) in high schools, Shanxi, China. **Table S7** Associations between microbial α-diversity (observed number of species) and rhinitis among students (N=1,332) in high schools, Shanxi, China. **Table S8** Associations between microbial α-diversity (observed number of species) and rhinoconjunctivitis among students (N=1,332) in high schools, Shanxi, China. **Table S9** Differentially abundant species between rural and urban high schools in Shanxi, China.

## Data Availability

The clean reads were deposited in the Genome Sequence Archive (https://bigd.big.ac.cn/gsa) in the National Genomic Data Center with the accession number CRA003476.
