# Supplementary figures and images for: Indoor bacterial, fungal and viral species and functional genes in urban and rural schools in Shanxi Province, China–association with asthma, rhinitis and rhinoconjunctivitis in high school students
Source: Microbiome. 2021 Jun 12;9:138. doi: 10.1186/s40168-021-01091-0 (PMC8199840; doi:10.1186/s40168-021-01091-0)

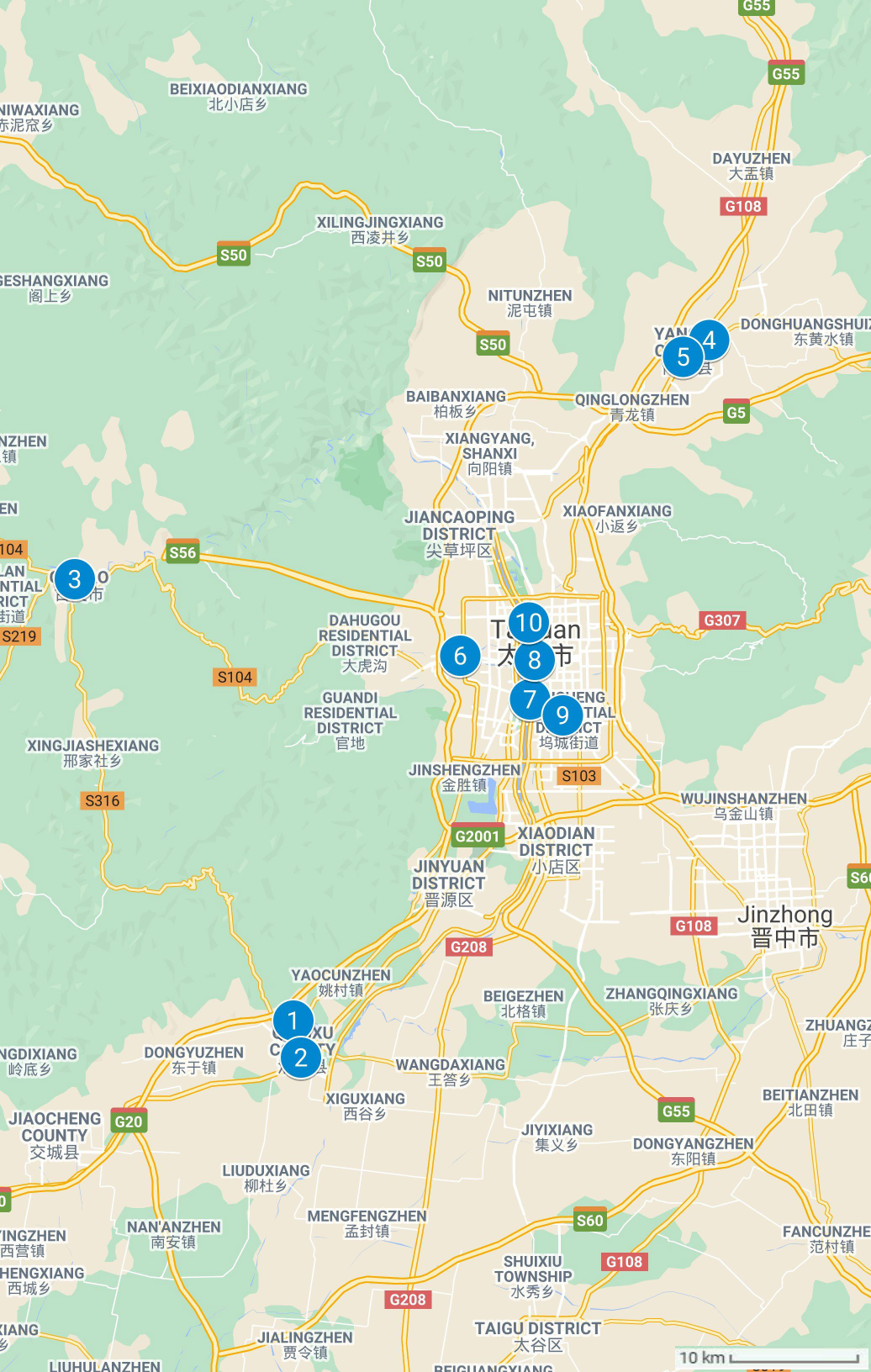

Supplement: Supplementary file 2 — Additional file 1: Figure S1. Locations of schools sampled in this study. [file 40168_2021_1091_MOESM2_ESM.pdf]

# The Rarefaction Curve

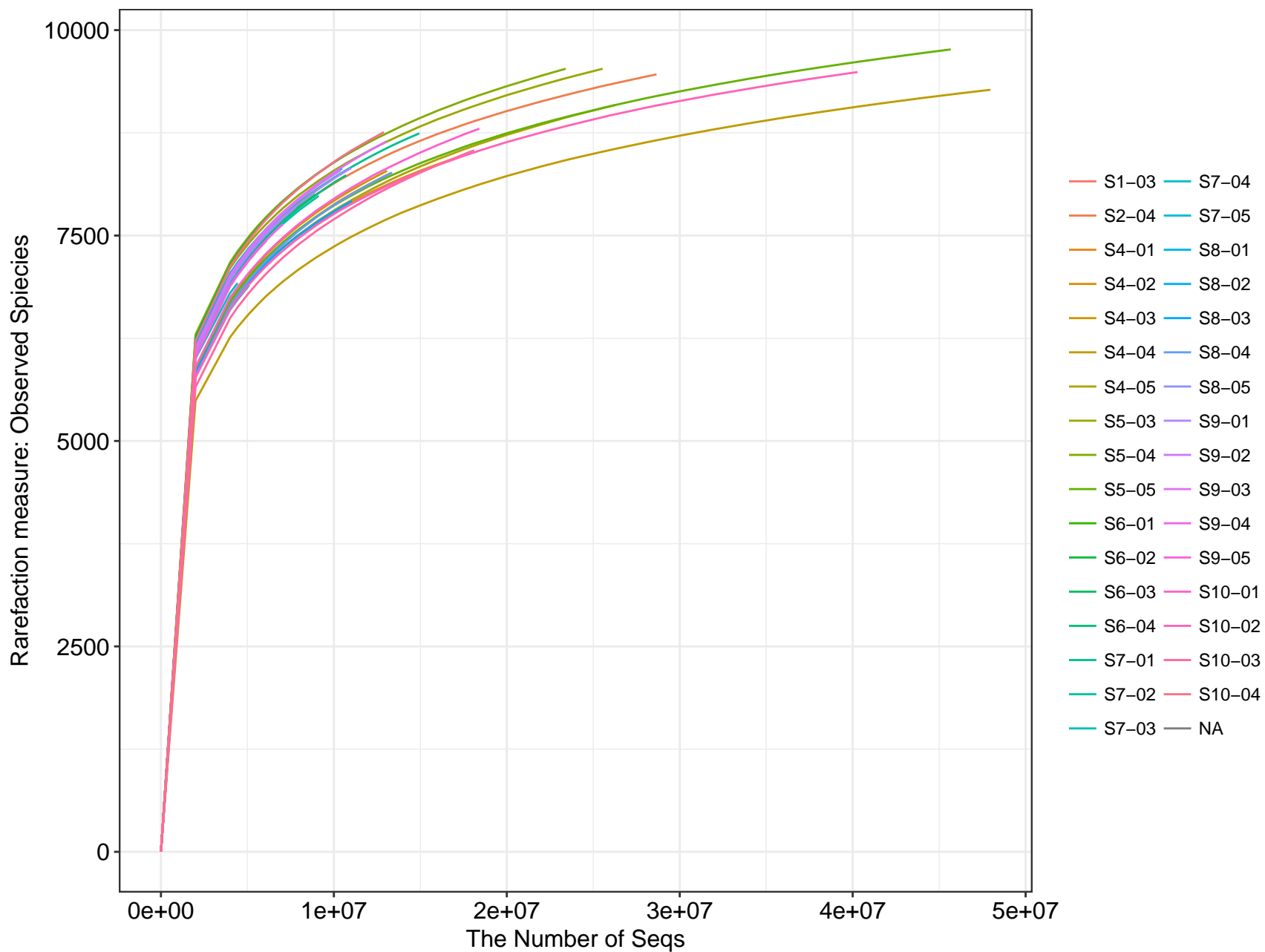

Supplement: Supplementary file 3 — Additional file 2: Figure S2. Rarefaction curve of observed species among all samples. [file 40168_2021_1091_MOESM3_ESM.pdf]
